# K‐Ras mutation and amplification status is predictive of resistance and high basal pAKT is predictive of sensitivity to everolimus in biliary tract cancer cell lines

**DOI:** 10.1002/1878-0261.12078

**Published:** 2017-06-14

**Authors:** Yvonne Yeung, David K. Lau, Fiona Chionh, Hoanh Tran, Janson W. T. Tse, Andrew J. Weickhardt, Mehrdad Nikfarjam, Andrew M. Scott, Niall C. Tebbutt, John M. Mariadason

**Affiliations:** ^1^ Olivia Newton John Cancer Research Institute Melbourne Australia; ^2^ Ludwig Institute for Cancer Research Melbourne‐Austin Branch Australia; ^3^ School of Cancer Medicine La Trobe University Melbourne Australia; ^4^ Department of Surgery Austin Health University of Melbourne Australia

**Keywords:** AKT, biliary tract cancer, everolimus, K‐Ras, mTOR

## Abstract

Advanced biliary tract cancer (BTC) has a poor prognosis and limited treatment options. The PI3K/Akt/mTOR signalling pathway is hyperactivated in a subset of BTCs, and clinical activity of the mTOR inhibitor everolimus has been observed in some patients with BTC. The goal of this study was to identify biomarkers predictive of everolimus response. Twenty BTC cell lines were assessed for everolimus sensitivity with a spectrum of growth inhibitory responses observed. Molecular biomarkers of sensitivity and resistance were identified by interrogation of the activation status of the Ras/MAPK and PI3K/Akt/mTOR pathways. K‐Ras mutations and/or amplifications were identified in 45% of cell lines and were associated with resistance to everolimus. Activating mutations in PIK3CA or loss of PTEN was not predictive of everolimus response; however, high basal levels of pAKT were associated with sensitivity, independent of Ras/MAPK pathway activation status. Notably, everolimus inhibited mTOR signalling to a similar extent in sensitive and resistant cell lines, suggesting that relative dependence on the mTOR pathway rather than the magnitude of pathway inhibition determines everolimus response. Consistent with the known limitations of rapalogs, everolimus induced feedback‐mediated activation of AKT in BTC cell lines, which could be overcome by cotreatment with an AKT inhibitor or ATP‐competitive mTORC1/mTORC2 inhibitors. However, both approaches failed to induce greater apoptosis compared to everolimus, and mTORC1/mTORC2 kinase inhibitors induced compensatory activation of pERK, identifying an inherent limitation of these agents in BTC cell lines. These findings suggest that future trials of everolimus in BTC would benefit from preselecting patients based on their K‐Ras and PI3K/mTOR pathway activation status. The study also identifies strategies for enhancing inhibition of the PI3K/mTOR pathway in BTC cell lines.

**Abbreviations**
BTC: biliary tract cancer
DCR: disease control rate
MAPK: mitogen‐activated protein kinase
ORR: overall response rate
RTK: receptor tyrosine kinase
S6K: S6 kinase

## 1 Introduction

Biliary tract cancers (BTCs) are a heterogeneous group of anatomically distinct adenocarcinomas, which include intra‐ and extrahepatic cholangiocarcinoma and gallbladder cancer that are extremely challenging to diagnose and treat. BTCs arise as a result of epithelial cells lining the biliary tract (cholangiocytes) undergoing dysplasia, hyperplasia, carcinoma *in situ*, and finally invasive carcinoma. The incidence of the disease varies around the world, with highest rates in north‐eastern Thailand and neighbouring Laos and Cambodia where liver fluke infestations (*Opisthorchis viverrini*) are endemic (Charbel and Al‐Kawas, 2011; Geynisman and Catenacci, 2012). In the United States, there are approximately 12 000 new cases of BTC annually, with the majority (80–90%) presenting with advanced disease (Charbel and Al‐Kawas, 2011; Hezel *et al*., 2010). The incidence of BTC, particularly intrahepatic cholangiocarcinoma, is increasing in the Western world for reasons that are presently unknown (Hezel *et al*., 2010). Systemic chemotherapy has only modest activity in the metastatic setting, with gemcitabine plus cisplatin the standard of care, providing a median overall survival of 12 months (Chan and Berlin, 2015; Valle *et al*., 2009). There is therefore an urgent need for new treatments for this disease.

One potential treatment target is the PI3K/AKT/mTOR pathway, which is activated in a subset of BTCs (Bhat *et al*., 2013 Bhat, 2013 #2923; Lee *et al*., 2012; Populo *et al*., 2012; Wu *et al*., 2007). This has been demonstrated immunohistochemically by the presence of overlapping positive p‐mTOR and pAKT staining in 30–50% of BTCs (Lee *et al*., 2012). Genetic alterations that constitutively activate this pathway also occur, although at low frequency. These include activating mutations in PIK3CA (4–7%) (Nakamura *et al*., 2015; Riener *et al*., 2008), inactivating mutations in PIK3R1 (4%), TSC1 (3%), TSC2 (1%) and amplifications in AKT3 (3%) (Nakamura *et al*., 2015). Finally, 5–30% of BTCs harbour mutations, amplifications and fusions in receptor tyrosine kinases of the Erb and FGFR families, and c‐MET (Hezel *et al*., 2010; Li *et al*., 2014; Nakamura *et al*., 2015; Voss *et al*., 2013), which can all activate signalling through the PI3K/AKT/mTOR pathway.

The canonical PI3K/AKT/mTOR pathway is initiated by ligand binding and activation of receptor tyrosine kinases (RTKs). Activated RTKs recruit and phosphorylate IRS‐1 causing activation of PI3K and subsequent activation of AKT. Activated AKT phosphorylates a number of substrates including TSC2, which prevents TSC1/TSC2 complex formation (Beauchamp and Platanias, 2013; S. Zhang and Yu, 2010). This results in activation of the small GTPase Rheb, which phosphorylates and activates mTOR within the mTORC1 complex (Beauchamp and Platanias, 2013). Among the best characterized substrates of mTORC1 are S6 kinase (S6K), which phosphorylates several residues on the S6 ribosomal protein and promotes protein synthesis. Likewise, mTORC1 induces phosphorylation of 4E‐BPs, enabling them to bind the 5' cap of mRNA and initiate protein translation. mTORC1 therefore is a major regulator of ribosome biogenesis and protein synthesis, through which it functions to drive cell growth. PI3K/AKT/mTOR signalling is also regulated by multiple negative feedback loops (Beauchamp and Platanias, 2013; Santoni *et al*., 2014). For example, S6K inhibits IRS‐1 at both the transcriptional and protein level. Inhibition of mTORC1 and its downstream target S6K consequently results in relief of this feedback inhibition and reactivation of IRS‐1 (O'Reilly *et al*., 2006). Importantly, S6K also inhibits the mTORC2 complex by phosphorylating RICTOR at Thr1135. Inhibition of mTORC1 and S6K consequently results in mTORC2 activation, which notably can activate AKT (Beauchamp and Platanias, 2013).

Based on the frequent upregulation of the PI3K/AKT/mTOR pathway in human cancer, and its role in driving tumour cell growth, several inhibitors have been developed which target either the mTORC1 complex or mTOR directly. Agents that target the mTORC1 complex include rapamycin (sirolimus) and its derivatives everolimus and temsirolimus (rapalogs). These are allosteric inhibitors that bind and form a complex with the intracellular protein FBP12, which interacts with mTORC1 and inhibits the activity of the complex. Of note, the FBP12/rapalog complex does not inhibit the mTORC2 complex. Consequently, due to relief of the feedback loops described above, rapalogs induce paradoxical activation of AKT (Beauchamp and Platanias, 2013; O'Reilly *et al*., 2006). Nevertheless, despite this limitation, everolimus (RAD001, Afinitor) and temsirolimus have clinical activity in advanced renal cell cancers, advanced pancreatic neuroendocrine tumours (PNET) and advanced hormone receptor‐positive/HER2‐negative breast cancers, with everolimus FDA‐approved for the treatment of these tumours (O'Reilly and McSheehy, 2010; Populo *et al*., 2012).

Inhibition of mTOR signalling has demonstrated antitumour activity in individual biliary tract cancer cell lines (Moolthiya *et al*., 2014) and in a transgenic mouse model of biliary tract cancer (Wu *et al*., 2007). Furthermore, two recent phase II clinical trials tested the safety and efficacy of everolimus in metastatic BTC. While response rates were low, some activity was noted. The ITMO study by Buzzoni *et al*. assessed the efficacy of single‐agent everolimus in 39 chemorefractory patients and reported an overall response rate (ORR) of 5%, a disease control rate (DCR) of 45% at 8 weeks and a median overall survival time of 7.7 months (Buzzoni *et al*., 2014), which compared favourably with previously evaluated chemotherapy‐based regimens (Walter *et al*., 2013). Comparatively, the phase II RADiChol trial performed at our institute assessed single‐agent everolimus activity in previously untreated patients and reported an ORR of 8%, a DCR of 56% at 12 weeks and a median overall survival of 9.5 months (Yeung *et al*., 2014).

While these studies have revealed the existence of a subset of patients with BTC who benefit from an mTOR inhibitor, there are currently no established biomarkers of response to these agents. The objective of this study was to identify biomarkers of everolimus response by *in vitro* screening of a panel of BTC cell lines. We specifically focussed on mutations in the PI3K pathway and readouts of pathway activity, as well as activation status of K‐Ras, as K‐Ras mutations are predictive of resistance to everolimus in colorectal cancer (Di Nicolantonio *et al*., 2010). We demonstrate that cell lines with K‐Ras mutations and/or gene amplifications were associated with resistance to everolimus, even in cell lines harbouring PIK3CA mutations or PTEN loss, suggesting that K‐Ras acts dominantly in this context. Conversely, in K‐Ras WT cell lines, high basal pAKT expression was associated with sensitivity, independent of K‐Ras status. In addition, we observed that everolimus inhibited downstream signalling to a similar extent in sensitive and resistant cell lines, suggesting that the degree of dependence of a cell line on mTOR signalling determines everolimus response, rather than the extent of pathway inhibition.

## 2 Methods

### 2.1 Cell culture

A panel of 20 BTC cell lines derived from distinct anatomical locations within the biliary tree were obtained from the following sources: HuH‐28, OZ, OCUG‐1, NOZ (HSRB – Japan Health Sciences Foundation), SNU‐1079, SNU‐1196, SNU‐245, SNU‐478, SNU‐869, SNU‐308 (KCLB – Korean Cell Line Bank), TKKK, TGBC2TKB, TGBC18TKB, TGBC14TKB, G‐415, HuCCT1 (RIKEN Bioresources Centre, Japan), SK‐ChA‐1, Mz‐ChA‐2 (Zurich University, A. Knuth), TFK‐1, EGI‐1 (DSMZ). STR profiling was performed on all lines using the Promega StemElite ID System at the Queensland Institute of Medical Research DNA Sequencing and Fragment Analysis Facility (Brisbane, Queensland, Australia), and all lines were found to be unique. All cell lines were maintained in Dulbecco's minimal essential media/F12 (DMEM/F12) supplemented with 10% FBS, 1% HEPES buffer and penicillin/streptomycin (50 U·mL^−1^ and 50 μg·mL^−1^, respectively), at 37 °C with 5% CO_2_. The anatomical location from which each cell line is derived is listed in Table 1.

**Table 1:** Anatomical location, PTEN expression and PIK3CA, BRAF and K‐Ras mutation status of the biliary tract cancer cell lines

Cell lines	Anatomical location	PIK3CA/PTEN	K‐Ras/BRAF^*V600E*^
HuH28	Intrahepatic	PIK3CA Mut‐E545K	WT/WT
OZ	Intrahepatic	WT	Mut–Q61K/WT
SNU‐1079	Intrahepatic	WT	WT/WT
TKKK	Intrahepatic	WT	WT/WT
SNU‐1196	Intrahepatic	WT	WT/WT
SK‐ChA‐1	Extrahepatic	WT	WT/WT
TFK‐1	Extrahepatic	WT	WT/WT
SNU‐245	Extrahepatic	WT	WT/WT
SNU‐478	Extrahepatic	WT	WT/WT
SNU‐869	Extrahepatic	PIK3CA Mut‐E545A	Mut‐G12D/WT
TGBC18TKB	Extrahepatic	WT	WT/WT
SNU‐308	Gallbladder	PTEN Null	WT/WT
TGBC14TKB	Gallbladder	PTEN Null	Mut‐G13C/WT
Mz‐ChA‐2	Gallbladder	WT	WT/WT
TGBC2TKB	Gallbladder (met)	PTEN Null	WT/WT
G‐415	Gallbladder (met)	WT	Mut‐G13D/WT
NOZ	Gallbladder (met)	WT	Mut‐G12V/WT
OCUG‐1	Gallbladder (met)	WT	WT/WT
HuCCT1	Biliary, ascites	WT	Mut‐G12D/WT
EGI‐1	Biliary, ascites	WT	Mut‐G12D/WT

### 2.2 Chemicals

Everolimus, MK‐2206, KU‐0063794, gemcitabine and BEZ‐235 were obtained from Selleck Chemicals (Houston, USA).

### 2.3 Assessment of cell proliferation and cell cycle kinetics

Cell proliferation was determined by MTS (3‐(4,5‐dimethyl‐2‐yl)‐5‐(3‐carboxymethoxyphenyl)‐2‐(4‐sulfophenyl)‐2H‐tetrazolium, inner salt) assay using the CellTiter 96^®^ AQ_ueous_ Non‐Radioactive Cell Proliferation Assay as per manufacturer's instructions (Promega, Madison, WI, USA). Cell cycle distribution was assessed by propidium iodide (PI) staining and FACS analysis as previously described (Mariadason *et al*., 2000). The FlowJo program (LLC, ORL, USA) was used to model the FACS data.

### 2.4 Validation of K‐Ras, BRAF, and PIK3CA mutation status

Genomic DNA was extracted from cell pellets using DNeasy Blood and Tissue Kit (Qiagen, Hilden, Germany). The mutation status of K‐Ras (codons 12, 13 and 61), BRAF (V600) and PIK3CA (exons 9 and 20) was validated in the 20 BTC cell lines by conventional Sanger sequencing. Primers used for amplification of exon 2 of K‐Ras were: F: AGGCCTGCTGAAAATGACTGAATA and R: CTGTATCAAAGAATGGTCCTGCAC, for exon 3, F: TAAAA GGTGCACTGTAATAATCC and R: TAAAAACTATAATTACTCCTTAATG. Primers used for the amplification of exon 15 of BRAF were F: AACACATTTCAAGCCCCAAA and R: GAAACTGGTTTCAAAATATTCGTT, for exon 9 of PIK3CA, F: GCTTTTTCTGTAAATCATCTGTG, and R: CTGAGATCAGCCAAATTCAGT and for exon 20 of PIK3CA, F: CATTTGCTCCAAACTGACCA and R: TACTCCAAAGCCTCTTGCTC (for codon 1023 mutation) and F: ACATTCGAAAGACCCTAGCC and R: CAATTCCTATGCAATCGGTCT (for codon 1047 mutation).

### 2.5 K‐Ras gene amplification

Quantitative PCR was performed on 10–30 ng of genomic DNA per reaction with Power SYBR Green on a ViiA7 Real‐Time PCR System (Applied Biosystems, Foster City, CA, USA) using the following primers as described previously (Misale *et al*., 2014): KRAS gDNA F: CTGAGCTCCCCAAATAGCTG, KRAS gDNA R: AGGTTAGGGCTAGGCACCAT, gD12S1595 chr12 F: GGGATCTTATGATGTGTCAGG and gD12S1595 chr12 R: ACTCTTGGTCTCAGTCTGCC. Fluorescence *in situ* hybridization (FISH) was performed using the ZytoLight SPEC KRAS/CEN 12 Dual Color Probe (Zytovision, Bremerhaven, Germany) per the manufacturer's instructions.

### 2.6 Ras activity assays

RAS GTPase activation was measured using a Ras GTPase chemiluminescent ELISA Kit (ab134640, Abcam, Cambridge, UK) per the manufacturer's instructions.

### 2.7 Western blotting

Cells were lysed in 1% NP‐40, 50 mm Tris/HCl pH 7.5, 150 mm NaCl, 0.5% sodium deoxycholate, 1 mm EDTA, cOmplete EDTA‐free Protease Inhibitor Cocktail (Roche/Sigma‐Aldrich, Sydney Australia), PhosSTOP phosphatase Inhibitor cocktail (Roche/Sigma‐Aldrich) and 3 m urea. 15–30 μg of total protein per sample was electrophoresed on NuPAGE™ 4–12% Bis/Tris gels and transferred onto PVDF membranes using iBlot transfer stacks (Invitrogen, Carlsbad, CA, USA). Antibodies used in western blot analysis were phospho‐Akt Ser473 (cat # 4051), AKT1 (2938), PTEN (9559), phospho‐S6 Ser240/244 (5364), S6 (2317), phospho‐4EBP1 T37/46 (2855), 4EBP1 (9644), beclin (3495), phospho‐ERK Thr202/Tyr204 (4370), ERK1/2 (9107) and phospho‐mTOR Ser2448 (2976) from Cell Signalling Technologies (Danvers, MA), β‐tubulin (ab6046, Abcam) and anti‐β‐actin (A5316, Sigma). Secondary antibodies used were fluorescent‐labelled goat anti‐mouse (IRDye(R) 680 CW, LI‐COR, 1 : 15 000) and goat anti‐rabbit (IRDye(R) 800 CW, LI‐COR, 1 : 15 000). Signal was detected using an Odyssey (R) Infrared Imaging System (LI‐COR Biotechnology, Lincoln, NE, USA), and fluorescent intensities were quantified using odyssey software, version 3.0.29 (LI‐COR Biotechnology).

### 2.8 Immunohistochemistry

A tissue microarray was constructed from formalin‐fixed, paraffin‐embedded cell blocks. Slides underwent microwave heat‐induced epitope retrieval in Tris/EDTA pH 9.0 followed by incubation with primary antibodies overnight at 4 °C. Sections were incubated with REAL EnVision‐HRP secondary antibody, diaminobenzidine substrate (Dako, Santa Clara, CA, USA), and counterstained with haematoxylin.

### 2.9 Statistical analyses

Groups were compared using unpaired *t*‐tests. Ras activity was log‐transformed and groups were compared using one‐way anova and *post hoc* Tukey–Kramer tests. Continuous variables were compared using Pearson's correlation coefficients.

## 3 Results

### 3.1 Identification of BTC cell lines sensitive and refractory to everolimus treatment *in vitro*

**Figure 1:**
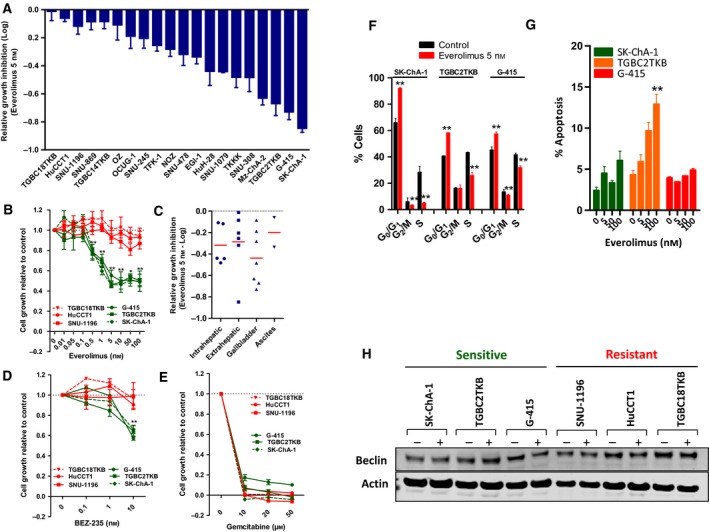
(A) Sensitivity of a panel of 20 biliary tract cancer cell lines to everolimus (5 nm). Cells were treated with everolimus for 72 h, and growth inhibition was determined using the MTS assay. (B) Response of the three most sensitive and resistant cell lines to a range of everolimus concentrations. Growth inhibition was determined by MTS assay 72 h post‐treatment. Values shown are mean ± SEM of a minimum of three independent experiments. (C) Sensitivity to everolimus according to anatomical location part of the biliary tract from which the cell lines were derived, or ascites. (D–E) Response of the three everolimus‐sensitive and resistant cell lines to (D) BEZ‐235 and (E) gemcitabine. Growth inhibition was determined by MTS assay 72 h post‐treatment. Values shown are mean ± SEM from a representative experiment with similar results observed in three biological replicates. Differences between the sensitive and resistant cell lines were compared using a *t*‐test, **P < 0.005. (F‐G) Effect of everolimus on cell cycle and apoptosis. (F) The three most sensitive BTC cell lines were treated with everolimus (5 nm) for 24 h and effects on cell cycle determined by propidium iodine staining and FACS analysis. (G) Effects on apoptosis were determined by PI staining after 72 h of treatment. Values shown are mean ± SD from a representative experiment. **P < 0.005. (H) Effect of everolimus on expression of the autophagy marker beclin. Cells were treated with everolimus (5 nm) for 48 h and beclin levels determined by western blot.

To characterize the response of BTC cell lines to everolimus, each of 20 BTC cell lines was treated with a range of everolimus concentrations (0.1–100 nm), and the effect on cell proliferation was assessed after 72 h using the MTS assay. Shown in Fig. 1A is the continuum of response of the cell lines to everolimus 5 nm, with G‐415, TGBC2TKB and SK‐ChA‐1 the three most sensitive and TGBC18TKB, HuCCT1 and SNU‐1196 the three most resistant cell lines in the panel (Fig. 1B). The differential response of these lines was consistently maintained across a range of everolimus concentrations, including the clinically relevant concentration of 50 nm (Tabernero *et al*., 2008). No difference in everolimus response and the anatomical location along the biliary tract from which the cell lines originated was observed (Fig. 1C). We next assessed the sensitivity of the three most sensitive and resistant cell lines to BEZ‐235, a dual PI3K/mTOR inhibitor. Cell lines sensitive to everolimus were also preferentially sensitive to BEZ‐235, suggesting that these lines are particularly dependent on mTOR signalling for their proliferation (Fig. 1D). In contrast, there was no difference in sensitivity to the chemotherapeutic agent gemcitabine, indicating that resistance to everolimus is not due to generic resistance of these cell lines to all treatments (*P* > 0.05, Fig. 1E).

To determine whether everolimus induced cytostatic or cytotoxic effects, changes in cell cycle kinetics and apoptosis following everolimus treatment were assessed in the three most sensitive lines. Everolimus induced a significant decrease in S phase with a concomitant increase in the percentage of cells in G_0_/G_1_ phase in all three lines (Fig. 1F). Assessment of apoptosis indicated that the effect of everolimus was mostly cytostatic, with apoptosis induction observed in only one (TGBC2TKB) of the three sensitive lines and only at higher concentrations (Fig. 1G). Finally, as everolimus has been reported to induce autophagy in other tumour cell lines (Ji *et al*., 2014; Thomas *et al*., 2012) and increases expression of the marker of autophagy, beclin‐1 (Ji *et al*., 2014), we examined its effect on beclin expression in BTC lines. In contrast to its effects in other tumour cells (Ji *et al*., 2014; Thomas *et al*., 2012), everolimus had no effect on beclin expression in either sensitive or resistant cell lines (Fig. 1H). Collectively, these findings demonstrate that everolimus predominantly mediates antiproliferative effects on BTC cells through the induction of G_0_/G_1_ cell cycle arrest.

### 3.2 Characterization of BTC cell lines for perturbations in the Ras/MAPK pathway

Having established the sensitivity of BTC lines to everolimus, we sought to identify biomarkers of response to this agent. We first focussed on the RAS/MAPK pathway as mutations in K‐Ras have been linked to resistance to everolimus in colorectal cancer (Di Nicolantonio *et al*., 2010), and genetic perturbations in the Ras/MAPK pathway have been documented in BTC (Li *et al*., 2014). Analysis of the mutation status of codons 12, 13 and 61 in K‐Ras identified mutations in 7 of the 20 lines (OZ, SNU‐869, TGBC14TKB, G415, NOZ, HuCCT1 and EGI‐1) (Table 1). No mutations in the V600 hotspot in BRAF were identified in any of the cell lines (Table 1).

**Figure 2:**
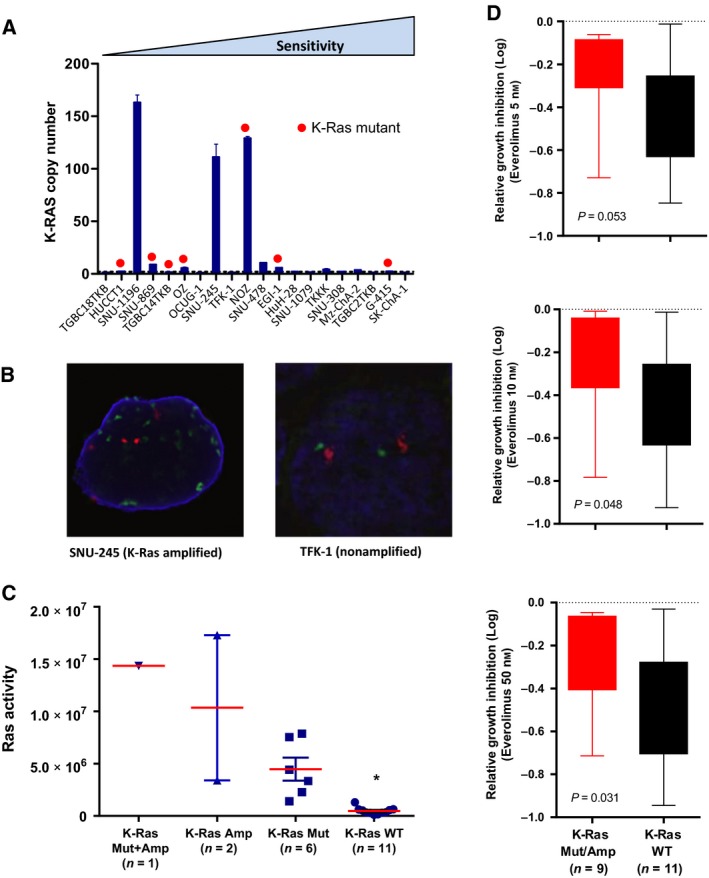
(A) K‐Ras gene copy number of BTC cell lines determined by quantitative PCR analysis of genomic DNA. (B) Representative FISH images of K‐Ras amplification status in SNU‐245 and TFK‐1 cells. CEP12 (red), K‐Ras (green) and DAPI (blue). (C) Ras activity of cell lines separated according to K‐Ras mutation and amplification status. Ras activity was assessed on cell lysates prepared from exponentially growing cells. *P < 0.05. (D) BTC cell lines were separated according to K‐Ras mutation and/or amplification status and sensitivity to everolimus at the 5, 10 and 50 nm doses compared using an unpaired *t*‐test.

In addition to mutations, K‐Ras gene amplifications occur in a subset of cholangiocarcinomas (Nakamura *et al*., 2015). We therefore determined the K‐Ras amplification status of each cell line by qPCR, identifying three amplified lines (SNU‐245, SNU‐1196 and NOZ) (Fig. 2A), of which NOZ also harboured a K‐Ras mutation. Amplification of K‐Ras was confirmed by fluorescence *in situ* hybridization using a dual colour K‐Ras/CEP12 FISH probe on all cell lines. Representative images of an amplified (SNU‐245) and nonamplified line (TFK‐1) are shown in Fig. 2B. To determine whether K‐Ras amplification resulted in increased Ras activity, we performed Ras activation assays across the 20 cell lines. Cell lines harbouring K‐Ras mutations or amplifications, alone or together, all exhibited high levels of Ras GTPase activity (*post hoc* Tukey–Kramer, *P* > 0.05), which were significantly greater that K‐Ras WT/nonamplified lines (*P* ≤ 0.01, *P* ≤ 0.001 and *P* ≤ 0.0001, respectively) (Fig. 2C).

### 3.3 Activating mutations or amplifications in K‐Ras confer resistance to everolimus

Having established the activation status of K‐Ras in the cell lines, we next examined associations with response. Separation of the cell lines as K‐Ras mutant/amplified versus WT demonstrated increased sensitivity of the WT lines at all three concentrations of everolimus examined, although the 5 nm group narrowly missed statistical significance (*P* = 0.053) (Fig. 2D).

### 3.4 Activation of the PI3K/PTEN/AKT pathway and everolimus response

**Figure 3:**
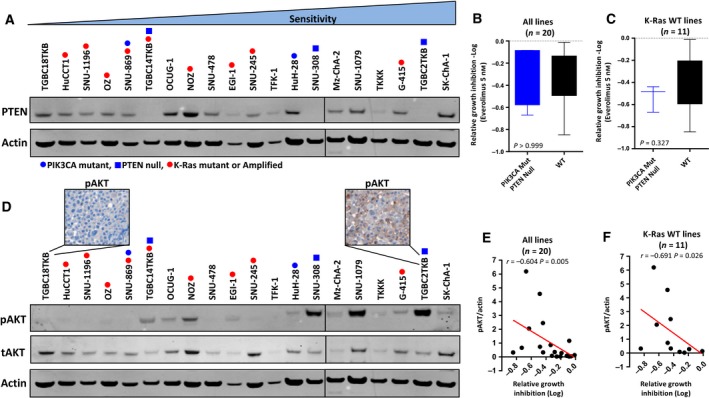
PI3K/PTEN/AKT pathway activation status and everolimus response. (A) PTEN protein expression levels in exponentially growing BTC cell lines assessed by western blot. (B) Dichotomization of all 20 cell lines as PIK3CA mutant/PTEN null or WT and association with everolimus response. (C) Dichotomization of the 11 K‐Ras WT cell lines as PIK3CA mutant/PTEN null or WT and association with everolimus response. (D) pAKT levels in exponentially growing BTC cell lines assessed by western blot and pAKT staining assessed by immunohistochemical staining of cell blocks generated from two representative cell lines. (E–F) Correlation of basal pAKT/tAKT levels with everolimus‐induced growth inhibition at the 5 nm concentration across (E) all 20 cell lines and (F) the 11 K‐Ras WT lines.

We next determined whether the activation status of the PI3K/PTEN/AKT pathway was predictive of everolimus response. Activating mutations in exons 9 and 20 of PIK3CA occur in a subset of BTCs and direct sequencing identified activating mutations in exon 9 in two cell lines, HuH‐28 and SNU‐869 (Table 1). In addition, as PI3K/AKT/mTOR pathway activation can also be mediated through PTEN loss, we examined PTEN protein expression by western blot and identified three cell lines with complete loss of PTEN expression (SNU‐308, TGBC14TKB and TGBC2TKB) (Fig. 3A).

However, separation of all lines as PIK3CA mutant/PTEN null or PIK3CA/PTEN WT revealed no difference in everolimus response (Fig. 3B). Notably, two of the lines harbouring a PI3K mutations or PTEN loss (SNU‐869 and TGBC14TKB) also harboured mutations in K‐Ras. Both of these lines were refractory to everolimus (Fig. 1), suggesting that Ras mutations are dominant over PI3K mutation or PTEN loss in determining everolimus response. As we had established that Ras/MAPK pathway mutations conferred resistance to everolimus, we examined the impact of activating mutations in PIK3CA or PTEN loss on everolimus response in the K‐Ras WT subset. While the PIK3CA mutant/PTEN null subset was on average more responsive to everolimus compared to the WT group, this difference was not statistically significant (Fig. 3C).

We next determined PI3K/AKT/mTOR pathway activation status by the assessment of basal pAKT levels by western blot across the cell lines, which revealed a range of expression levels (Fig. 3A). High basal pAKT levels in cell lines were associated with significantly greater growth inhibition in response to everolimus (*r *=* *−0.604, *P *=* *0.007, Fig. 3E). Importantly, this association remained statistically significant when only K‐Ras WT cell lines were considered (*r *=* *−0.691, *P *=* *0.026) (Fig. 3F).

### 3.5 Everolimus inhibits p‐mTOR and downstream signalling in sensitive and resistant cell lines

**Figure 4:**
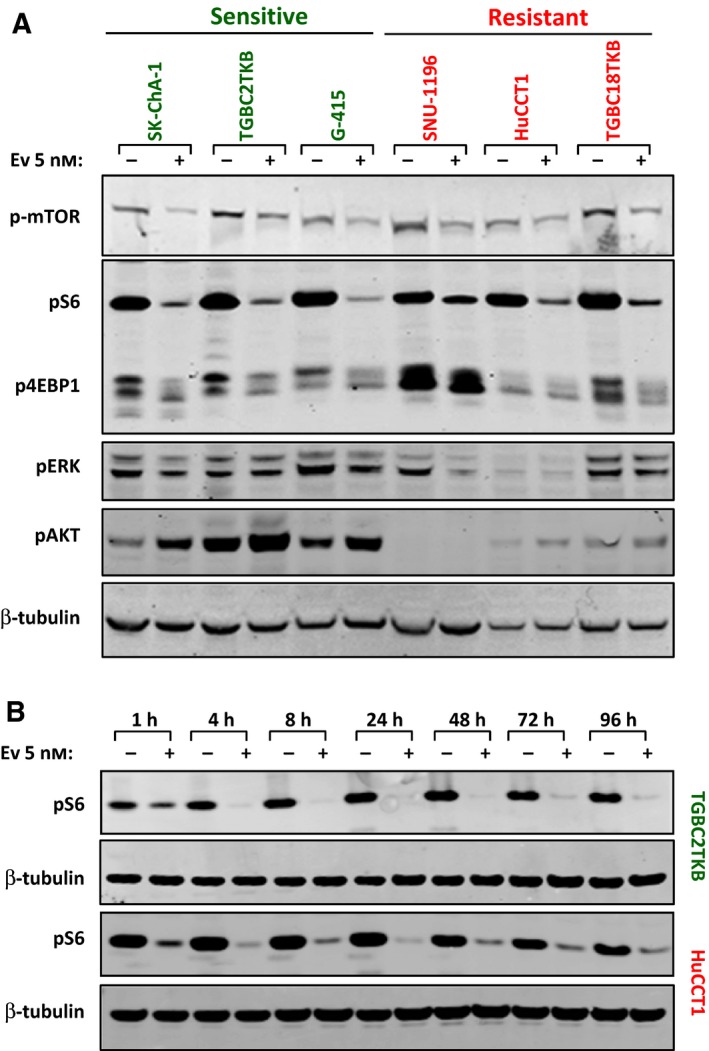
(A) Effect of everolimus on mTOR signalling in sensitive and resistant cell lines. The three most sensitive and resistant cell lines were treated with everolimus (5 nm) for 48 h and changes in p‐mTOR, pS6, p4EBP1, pAKT and pERK determined by western blot. (B) Extended time course analysis of the effect of everolimus on pS6 expression in a representative sensitive (TGBC2TKB) and resistant (HuCCT1) BTC cell line. Cells were treated with everolimus (5 nm) for 1–96 h and pS6 (Ser240/244) levels determined by western blot.

To determine whether the differential sensitivity to everolimus was also linked to the magnitude of inhibition of the mTOR pathway, we compared changes in p‐mTOR and downstream signalling components in the three most sensitive and resistant cell lines. Everolimus decreased p‐mTOR, pS6 and p4EBP1 in all cell lines after 48‐h treatment (Fig. 4A). Densitometry analysis revealed no significant differences in the magnitude of change in these markers between sensitive and resistant cells (*P* > 0.05 for p‐mTOR, pS6 and p4EBP1). Everolimus had no effect on pERK in any of the cell lines, confirming its specificity for targeting the PI3K/AKT/mTOR pathway, and lack of compensatory activation of this alternate signalling pathway.

We next asked whether there were differences in the duration of pathway inhibition following everolimus treatment of sensitive and resistant cells. Treatment of the sensitive (TGBC2TKB) and resistant (HuCCT1) cell lines with everolimus for 96 h resulted in strong suppression of pS6 in both cell lines, which remained suppressed over the duration of the time course, indicating that the differential sensitivity is not due to differences in the duration of pathway inhibition (Fig. 4B). Collectively, these findings demonstrate that everolimus inhibits downstream signalling in both sensitive and resistant lines and that the magnitude of pathway inhibition is not a key determinant of response.

Finally, a known limitation of rapalog treatment is feedback‐mediated activation of AKT (Sarbassov *et al*., 2005). As reported in other cell lines, everolimus increased pAKT levels in all three sensitive BTC cell lines (Fig. 4A).

### 3.6 Dual targeting of mTOR and AKT enhances pathway inhibition and cell growth inhibition

**Figure 5:**
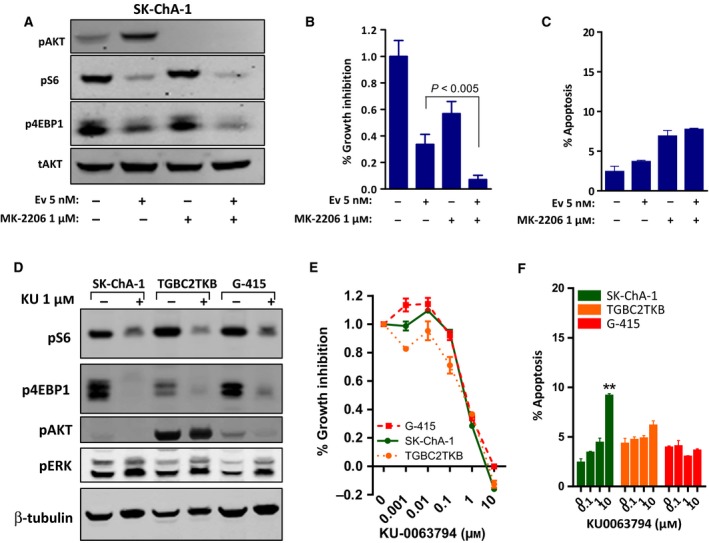
(A–C) Effect of combination treatment with everolimus and the AKT inhibitor MK‐2206 in SK‐ChA‐1 cells. (A) Cells were treated with everolimus (5 nm) or MK‐2206 (1 μm) alone or in combination for 24 h. (B–C) Cells were treated with everolimus and MK‐2206, alone and in combination for 72 h, and (B) cell proliferation was assessed using the MTS assay and (C) apoptosis determined by propidium iodide staining and FACS analysis. Values shown are mean ± SEM from a representative experiment with similar results observed in three biological replicates. (D–F) Response of BTC cell lines to the ATP‐competitive mTOR inhibitor KU‐0063794. (D) Cells were treated with KU‐0063794 (1 μm) for 24 h. (E) Cells were treated with KU‐0063794 for 72 h, and growth inhibition was assessed using the MTS assay. Values shown are mean ± SEM from a representative experiment with similar results observed in three biological replicates. (F) BTC cells were treated KU‐0063794 for 72 h, and apoptosis was determined by propidium iodide staining and FACS analysis. Values shown are mean ± SEM from a representative experiment, **P <* *0.005, *t*‐test.

Given the finding that everolimus induces feedback‐mediated activation of pAKT in sensitive cell lines, we asked whether blockade of pAKT could enhance the activity of everolimus. To address this, sensitive Sk‐ChA‐1 cells were cotreated with everolimus and the AKT inhibitor, MK‐2206. MK‐2206 markedly attenuated basal and everolimus‐induced pAKT, as well as the downstream signalling components pS6 and p4EBP1. Consistent with these effects on cell signalling, combination treatment inhibited cell proliferation to a greater extent than either agent alone (Fig. 5A,B). Assessment of the effect on apoptosis revealed a modest but statistically significant increase following MK‐2206 treatment alone; however, no further increase was observed following combination treatment (Fig. 5C).

An alternate strategy for circumventing pAKT activation following mTORC1 inhibition is the use of ATP‐competitive inhibitors of mTORC1 and mTORC2. These agents also effectively inhibit p4EBP1 in cells in which everolimus is unable to do so (Fan *et al*., 2016; Y. Zhang and Zheng, 2012). We therefore tested the effect of KU‐0063794 on downstream signalling in the everolimus‐sensitive lines. KU‐0063794 induced robust inhibition of the downstream signalling readouts pS6 and p4EBP1 without inducing activation of pAKT in all three cell lines. However, in contrast to everolimus, KU‐0063794 induced pERK levels in all three cell lines, indicating that ATP‐competitive inhibitors induce compensatory activation of this alternate signalling pathway in BTC cells (Fig. 5D).

Assessment of the effects on cell proliferation demonstrated strong inhibition of cell growth in all three cell lines (Fig. 5E). However, as observed for everolimus, KU‐0063794 only induced modest apoptosis in one of the three cell lines (Fig. 5F), indicating that prevention of feedback‐mediated activation of pAKT is not sufficient to induce apoptosis following mTOR inhibition in BTC cells.

## 4 Discussion

Two recent early‐phase clinical trials have demonstrated that a subgroup of patients with biliary tract cancer gain benefit from treatment with the mTOR inhibitor everolimus (Buzzoni *et al*., 2014; Yeung *et al*., 2014). To identify biomarkers that can enable *a priori* identification of this patient subgroup, we screened a panel of 20 BTC cell lines for everolimus response and, consistent with the clinical findings, identified a subset of cell lines sensitive to this agent. The effect of everolimus on these lines was largely cytostatic, inducing G_0_/G_1_ cell cycle arrest. This finding is consistent with the effect of this agent on other cell lines (Breuleux *et al*., 2009).

Our analyses identified a positive and a negative predictor of everolimus response. First, we observed an association between K‐Ras mutation and amplification status and resistance to everolimus. Examination of this link was motivated by the findings of a phase II study of single‐agent everolimus in colorectal cancer (Di Nicolantonio *et al*., 2010), and response of colon cancer cell lines to the ATP‐competitive mTOR inhibitor PP242 (Ducker *et al*., 2014), which demonstrated an association between K‐Ras mutation status and resistance to mTOR inhibitors. Similarly, a study in a large panel of ovarian cancer cell lines identified a link between K‐Ras/BRAF mutation status and resistance to the dual PI3K/mTOR inhibitor, PF‐04691502 (Sheppard *et al*., 2013). The current findings therefore extend this observation by establishing K‐Ras mutation and amplification status as an informative predictor of everolimus response that transcends tumour type.

Notably, in this study, we identified 3/20 BTC cell lines that harboured K‐Ras gene amplification and 7/20 lines with K‐Ras hotspot mutations (and one cell line with both). While K‐Ras mutations and amplifications do occur in primary BTC, the collective frequency of these events in the cell line panel (45%) is significantly greater than that observed in primary cancers (18%) (Nakamura *et al*., 2015), suggesting that tumours harbouring these genetic alterations may be more amenable to cell line generation.

In addition to establishing K‐Ras mutation and amplification status as a marker of resistance, our findings also established high basal expression of pAKT as a positive predictor of everolimus sensitivity. This finding is consistent with two prior *in vitro* analyses of similar numbers of cell lines derived from a range of tumour types (Breuleux *et al*., 2009;O'Reilly and McSheehy, 2010). Similarly, immunohistochemical analysis of 19 patient tumour samples from a randomized phase 2 trial assessing the activity of temsirolimus in renal cell cancer identified a trend between positive expression of pAKT and response, although this narrowly missed statistical significance (*P* = 0.07) (Cho *et al*., 2007). Collectively, we interpret these findings to suggest that cell lines with high pAKT have greater dependence on PI3K/AKT/mTOR signalling for their proliferation as evidenced by their greater sensitivity to pathway inhibition. Conversely, lines harbouring K‐Ras mutations and/or amplification may be more dependent on the Ras/MAPK pathway and are consequently less responsive to mTOR inhibition.

We also investigated whether everolimus response was dependent on the magnitude or duration of mTOR pathway inhibition. These analyses demonstrated that everolimus inhibited downstream signalling to a similar extent in sensitive and resistant cells, indicating that differential pathway blockade was not the primary determinant of sensitivity. Instead, they support the conclusion that cell lines inherently dependent on mTOR signalling, reflected by high basal pAKT levels, are preferentially sensitive to pathway inhibition, while cell lines dependent on the Ras/MAPK pathway are less responsive.

Consistent with the known limitation of rapalogs, which has been observed in several tumour types (Yeung *et al*., 2014), everolimus induced feedback‐mediated activation of pAKT in BTC cell lines. Several mechanisms by which this occurs have been described, including inhibition of mTORC1 reducing S6‐mediated phosphorylation of RICTOR, a functional repressor of mTORC2, and consequent mTORC2‐mediated activation of pAKT (Santoni *et al*., 2014). These findings suggested that by inhibiting the feedback‐mediated activation of pAKT, sensitivity to everolimus may be enhanced. This was confirmed by the demonstration that dual treatment with everolimus and the pAKT inhibitor, MK2206, resulted in greater inhibition of downstream signalling and cell proliferation. In addition, while dual targeting of mTOR1 and AKT represents one approach of circumventing feedback‐induced pAKT activation, agents that directly inhibit the kinase activity of both mTORC1 and mTORC2 are also being developed, with several agents in early‐phase clinical testing (Santoni *et al*., 2014; Schenone *et al*., 2011). Indeed, treatment of everolimus‐sensitive BTC cell lines with the ATP‐competitive TORC1/TORC2 inhibitor KU‐0063794 (Garcia‐Martinez *et al*., 2009) resulted in robust inhibition of downstream signalling without paradoxical activation of pAKT. Notably, however, neither of these approaches was sufficient to induce greater levels of apoptosis compared to everolimus alone, indicating that feedback‐mediated activation of pAKT is not the basis for failure of everolimus to induce apoptosis.

A further observation made during these studies was the feedback‐mediated activation of pERK in BTC cells following treatment with ATP‐competitive TORC1/TORC2. Similar findings have previously been reported in pancreatic cancer cells (Soares *et al*., 2013) and underscore that while these agents can overcome some of the limitations of rapalogs, they contain other inherent limitations. The relative activities of everolimus versus ATP‐competitive TORC1/TORC2 inhibitors will therefore require careful clinical comparison.

In summary, our findings establish a significant association between high basal pAKT expression and sensitivity to everolimus and K‐Ras mutations/amplifications with resistance. An implication of these findings is that subsequent trials of everolimus for this indication may benefit from enrichment of patients according to K‐Ras mutation/amplification status and pAKT expression. Our findings also indicate that while therapeutic targeting of the PI3K/AKT/mTOR pathway using combinations of everolimus with AKT inhibitors or ATP‐competitive mTOR inhibitors that inhibit both mTORC1 and mTORC2 represents strategies for more robust pathway inhibition, they do not induce greater levels of cell death and present their own inherent limitations.

## Author Contributions

The study was conceived by AJW, MN, AMS, NCT and JMM. Experiments and data analysis were performed by YY, DL, FC, HT, JT and JMM. All authors were involved in writing and approval of the manuscript.
